# Supplementary material for: SMURF1-Induced Ubiquitination of FTH1 Disrupts Iron Homeostasis and Suppresses Myogenesis
Source: Int J Mol Sci. 2025 Feb 6;26(3):1390. doi: 10.3390/ijms26031390 (PMC11818545; doi:10.3390/ijms26031390)
Supplement: Supplementary file 1 [file ijms-26-01390-s001.zip › ijms-3389752-supplementary.pdf]

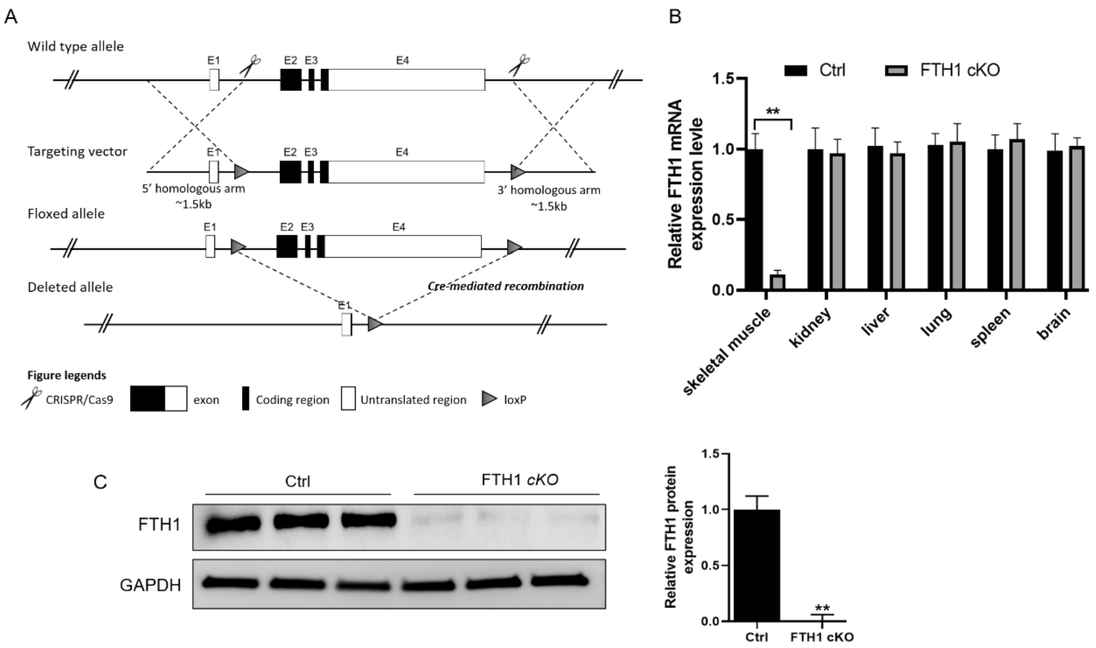

Figure S1. Generation of FTH1 knockout mice. (A) Schematic diagram of the FTH1 gene knockout strategy. (B) qPCR analysis of FHT1 mRNA expression levels in mouse tissues from FTH1 cKO and control mice. (C) Western blot analysis of FHT1 protein expression levels in mouse tissues from FTH1 cKO and control mice. Data are reported as means ± standard error of the mean (s.e.m.). * indicates $P < 0.05$; ** indicates $P < 0.01$.

Table S1. The primers used in this study.

Genes	Forward Primer (5′-3′)	Reverse Primer (5′-3′)
SMURF1	AGGCTCTGCAAGGCTCTAC	TTCACTCCACTGCAAAGCCA
MyoG	ACTCCCTTACGTCCATCGTG	CAGGACAGCCCCACTTAAAA
MyoD	AGCACTACAGTGGCGACTCA	GGCCGCTGTAATCCATCA
FTH1	TTCACTCCACTGCAAAGCCA	TCTCCCAGTCATCACGGTCT
ACSL4	CTTCCTCTTAAGGCCGGGAC	TCTCTTTGCCATAGCGTTTTTAG
COX2	CTTCGGGAGCACAACAGAGT	AAGTGGTAACCGCTCAGGTG
GAPDH	TGCCAAGTATGATGAACATCAAGAAG	GGTCCTCAGTGTAGCCCAAGAT
